# Combined treatment with mesenchymal stem cells and therapeutic hypothermia for neonatal hypoxic ischemic encephalopathy: a phase 1/2 randomized trial

**DOI:** 10.1038/s41598-025-98504-z

**Published:** 2025-05-10

**Authors:** Kazuko Wada, Akihito Takeuchi, Yoshinori Katayama, Natsuki Ohkawa, Masato Kantake, Kazumichi Fujioka, Toshiya Nishikubo, Yutaka Yamamoto, Yasumasa Yamada, Seiji Yoshimoto, Kiyoaki Sumi, Tomoaki Ioroi, Takeo Mure, Norihisa Wada, Yukimichi Nakano, Naoko Takasao, Kenji Tada, Tatsuyoshi Yamamoto, Hideaki Hirai, Yuji Sato, Hideyuki Ide, Satoshi Kusuda

**Affiliations:** 1 Department of Neonatal Medicine, Osaka Women’s and Children’s Hospital, 840 Murodo-cho, Izumi, Osaka 594-1101 Japan; 2 Division of Neonatology, NHO Okayama Medical Center, Okayama, Japan; 3 Department of Neonatology, Takatsuki General Hospital, Osaka, Japan; 4 Department of Neonatology, Juntendo University Shizuoka Hospital, Shizuoka, Japan; 5 Division of Neonatology, Juntendo University Nerima Hospital, Tokyo, Japan; 6 Department of Pediatrics, Kobe University Graduate School of Medicine, Hyogo, Japan; 7 Division of Neonatal Intensive Care, Fetal and Neonatal Medical Center, Nara Medical University Hospital, MaternalNara, Japan; 8 Department of Neonatology, Gifu Prefectural General Medical Center, Gifu, Japan; 9 Department of Perinatal and Neonatal Medicine, Aichi Medical University, Aichi, Japan; 10 Department of Neonatology, Hyogo Prefectural Kobe Children’s Hospital, Hyogo, Japan; 11 Department of Pediatrics, Aizenbashi Hospital, Osaka, Japan; 12 Department of Pediatrics, Perinatal Medical Center, Himeji Red Cross Hospital, Hyogo, Japan; 13 Department of Pediatrics, Chibune General Hospital, Osaka, Japan; 14 Kindai University Hospital Perinatal Medical Center, Osaka, Japan; 15 Research Division, JCR Pharmaceuticals, Hyogo, Japan; 16 Development Division, JCR Pharmaceuticals, 3-19 Kasuga-cho, Ashiya, Hyogo 659-0021 Japan; 17 Neonatal Research Network of Japan, Shinjuku Park Tower N30, 3-7-1, Nishishinjuku, Shinjuku, Tokyo 163-1030 Japan

**Keywords:** Neonatal brain damage, Neonatal brain damage, Stem-cell therapies

## Abstract

Neonatal hypoxic ischemic encephalopathy (nHIE) is a serious disease that causes severe and chronic neurological damage. Hypothermia therapy improves patients’ outcomes albeit with some limitations, but combining it with treatment with cord blood cells (analogous to mesenchymal stem cells [MSCs]) reportedly improves its effectiveness. TEMCELL HS Inj. (Temcell), a human bone marrow-derived MSC product used for acute graft-versus-host disease, seems an appropriate candidate for this combination therapy. Therefore, we performed a randomized, parallel-group study to compare combined treatment with Temcell and hypothermia versus hypothermia therapy-alone to evaluate the safety and efficacy of Temcell in nHIE patients. The primary endpoint was treatment response defined as an overall developmental quotient of ≥ 85 at 18 months of age. Fourteen patients were enrolled and randomized, with 7 assigned to each group. Both groups had similar demographic characteristics and nHIE severity. Treatment response was observed in 4 of the 6 (66.7%) patients in the Temcell combination group, and in 4 of the 7 patients (57.1%) in the hypothermia therapy-alone group. No marked differences in safety profile were observed between the groups. These results indicate that the efficacy of Temcell combined with hypothermia is comparable to therapeutic hypothermia for patients with nHIE.

Clinical Trial Registration: jRCT1080224818.

**Supplementary Information:**

The online version contains supplementary material available at 10.1038/s41598-025-98504-z.

## Introduction

Hypoxic ischemic encephalopathy (HIE) is a severe and chronic central nervous system (CNS) disorder caused by hypoxic ischemia. Hypoxia not only causes nerve cell death (primary nerve cell necrosis) due to excitotoxicity, edema, and necrosis, but also helps release intracellular factors that activate microglia and macrophages and induce inflammatory reactions^1,2^. HIE in neonates (nHIE) is associated with decreased cerebral blood flow during pregnancy or delivery as well as inflammatory disorders, damaging the CNS and leading to multifaceted clinical manifestations (e.g. motor and neurocognitive impairments and cerebral palsy).

Therapeutic hypothermia has been reported to improve neurological outcomes in patients with nHIE by slowing the brain metabolism via lowered body temperature, thereby mitigating brain damage through suppression of neuronal depolarization and excessive calcium influx into cells^3^. However, the prognosis of nHIE remains extremely poor, with approximately 45% of patients treated with therapeutic hypothermia dying or developing serious sequelae^4^; furthermore, one study showed that 2-year cognitive outcomes in patients treated with therapeutic hypothermia were no better than those in patients who survived HIE without hypothermia^5^. Hence, development of an effective treatment has long been awaited.

Treatment with bone-marrow derived mesenchymal stem cells (MSCs) has been reported to improve cognitive and motor skills in animal models of hypoxic encephalopathy^6–9^. It can be assumed that the differentiation of MSCs into neurons, oligodendrocytes and other glial cells helps repair neuronal damage in HIE^10^, and that their paracrine and trophic actions are effective against hypoxic-ischemic injury^11^. Intravenous transfusion of MSC in patients with chronic stroke has been shown safe with behavioral gains^12^, while Cotten et al. have demonstrated that cord blood cell administration, analogous to MSC administration, combined with hypothermia is more effective than hypothermia alone in treating patients with nHIE^13^. However, further examination of the safety and efficacy of this new combination therapy for nHIE is required.

Human mesenchymal stem cells (hMSCs) are undifferentiated cells in the bone marrow that are considered to be hypoimmunogenic. When clinically needed, pre-prepared third party-derived hMSCs can be promptly administered irrespective of human leukocyte antigen compatibility. TEMCELL HS Inj. (hereafter Temcell), an hMSC product derived from nucleated cells in healthy adult bone marrow fluid, has been shown to increase production of anti-inflammatory cytokines and to inhibit T cell proliferation. Transplantation of bone marrow-derived MSCs has been shown to decrease CNS damage and improve motor and cognitive functions in animal models of hypoxic cerebral ischemia thanks to the migratory ability of MSCs, which enables cytoprotective and anti-inflammatory actions^6–9^. Temcell was developed and approved in Japan in 2015 for general clinical use for the treatment of steroid-refractory acute graft-versus-host disease (GVHD) following hematopoietic stem cell transplantation (HSCT)^14–16^.

To address the aforementioned need to substantiate the safety and efficacy of MSC administration in combination with hypothermia as a new treatment for nHIE, a clinical study of this combination therapy was conducted in patients with nHIE in combination with hypothermia.

## Methods

### Study design

This was a multicenter, randomized, parallel-group phase I/II comparative study conducted in Japan between September 2019 and March 2022 (jRCT1080224818). The study protocol was in accordance with the ethical guidelines of the Declaration of Helsinki and was approved by the institutional review boards of all the investigational sites respectively. Written informed consent was obtained from the patients’ guardians (legally acceptable representatives) before any trial-related action was taken.

### Patients

In consideration of the feasibility of efficacy assessment and the number of institutions ready to enroll patients and provide the treatment, the sample size for the Temcell combination group and the hypothermia therapy-alone group were both set at 6. Over-enrollment was allowed to corroborate data collection.

The block randomization method was used to randomly assign patients in a 1:1 ratio to either a combined treatment group with Temcell and hypothermia therapy (Temcell combination group) or a hypothermia therapy-alone group; Thompson scores of ≥ 11 or < 11 were used as a stratification factor. A total of 14 patients were enrolled with 7 patients assigned to each group, while the total target sample size was 12 patients. The evaluations of the study results were conducted by central assessors blinded to the randomization and external to the investigational sites.

Inclusion criteria included a diagnosis of nHIE treated with hypothermia therapy (whole-body cooling only) according to the Japanese guidelines for hypothermia therapy for nHIE, and written informed consent provided by the patients’ guardians (legal representatives). Exclusion criteria included congenital anomalies (e.g. chromosome anomaly, congenital heart disease, congenital malformation syndrome); severe intracranial hemorrhage as detected by cranial ultrasonography or computed tomography (CT); severe infection (e.g. sepsis); hyperkalemia; a history of serious drug allergy or hypersensitivity in the parents; treatment of the mother with another investigational drug or product within 10 months before delivery of the infant; positive findings in the mother for hepatitis B surface (HBs) antigens, hepatitis C virus (HCV) antibody, human immunodeficiency virus (HIV) antibody, or syphilis during pregnancy; determination by the principal investigator or a sub-investigator that the patient was not suitable for the study.

### Treatment schedule

The first dose of Temcell was administered within 12–36 h after birth when the core body temperature had been stabilized at 33–34 °C under hypothermia therapy. Diluted Temcell (2.0 × 10^6^ cells of Temcell per 1 kg of body weight with 18 mL of saline per bag) was slowly infused intravenously over 10 min. As when used to treat acute GVHD after HSCT, Temcell was administered twice a week for 4 weeks, with an interval of at least 3 days between each dosing (Supplementary Figure S1). To prevent allergic reactions, adrenocortical hormone preparations were administered, if necessary, 30 min to 1 h before the start of each Temcell administration.

Because this study was the first time Temcell had been administered to neonates, the patients remained hospitalized during treatment to ensure their safety; the principal investigator or sub-investigator, along with other medical professionals, continually assessed the safety of continuing treatment.

### Evaluation

To evaluate motor and neurocognitive developmental disorders as the main clinical symptoms caused by CNS damage in patients with nHIE, the primary endpoint was neurocognitive development at 18 months of age, as assessed with the Kyoto Scale of Psychological Development 2001 (KSPD), a standardized assessment battery widely used in Japan for neurocognitive development in children from 0 years of age and adults^17,18^; KSPD has been shown to correlate well with the Bayley Scales of Infant Development, Third edition (BSID-III)^19,20^. An overall developmental quotient of ≥ 85 was deemed to indicate that treatment was effective (treatment-responsive), and the Temcell combination therapy was to be considered superior to hypothermia therapy alone if the percentage of treatment-responsive patients in the Temcell combination group exceeded that of the hypothermia therapy-alone group.

As for the secondary endpoints, the following items were assessed for each group: (1) BSID-III; (2) abnormalities in brain magnetic resonance imaging (MRI) scans (grades: 0, 1A to 2B, 3)^21^ taken at 10 days and 18 months after the first administration; (3) Gross Motor Function Classification System (GMFCS) classification (grades I to V) at 12 and 18 months; (4) Thompson score (summary statistics was calculated for the results [total points] of Thompson score measurements conducted at days 1, 2, and 3; (5) presence of epileptic seizures; (6) experience of gastrostomy; (7) experience of ventilation with tracheostomy; (8) use of inotropic agents; (9) number of days on artificial ventilation; (10) general condition (e.g. appetite, need for physical support). The final overall efficacy assessment was performed by an independent case review committee whose members were blinded to the randomization process.

Safety was assessed in terms of adverse events (AEs) and adverse drug reactions, laboratory data, percutaneous oxygen saturation, chest radiography, electrocardiography, echocardiography, echoencephalography. These were classified and listed according to system organ class (SOC) and preferred term (PT), as set out in the Japanese version of the Medical Dictionary for Regulatory Activities (MedDRA/J) (Ver. 25.1).

### Statistical analysis

All patients enrolled and, for the Temcell combination group, those administered at least once with Temcell were defined as the full analysis set and assessed for efficacy. The safety analysis set consisted of all patients enrolled. Descriptive statistics were used to summarize demographic and baseline clinical characteristics, which included gestational week, birth weight, Apgar score^22^, core body temperature on arrival at the clinical site, age of mother, sex, Sarnat classification^23^, transportation from another hospital, neonatal resuscitation, neonatal diagnosis, perinatal history, medication status during pregnancy, blood gas, and complications.

Missing data for the primary and secondary endpoints were not imputed using statistical methods. However, when the measurement values were below the quantitation limit, the values used for the summary statistics were determined by the case review committee. All analyses were performed with SAS software version 9.4 for Windows.

## Results

Fourteen patients fulfilled the inclusion and exclusion criteria and were enrolled across 9 investigational sites in Japan and randomized. All 7 patients assigned to the Temcell combination group received Temcell according to the study schedule. Among the 14 patients, 13 completed the study (one patient in the Temcell combination group was excluded due to protocol deviation) and were assessed for efficacy. Five of the 6 remaining patients in the Temcell combination group received adrenocortical hormone preparations. All 14 patients were assessed for safety. The demographic characteristics of both groups were generally similar (Table 1). The details of individual patient characteristics are summarized in Table 2. All patients were diagnosed as neonatal asphyxia and required cardiopulmonary resuscitation. On the Sarnat classification, a high proportion of the patients in both groups were classified as stage 2, but 1 patient in each group was stage 3. No marked differences were noted in treatment response in relation to stages. The median Apgar score at 5 min after birth was 4.0 in the Temcell combination group, and 3.0 in the hypothermia therapy-alone group, but one patient in the Temcell combination group had a score of 0. Maternal characteristics differed between the groups, with 66.7% of the mothers of patients in the Temcell combination group being multiparous and 83.3% having a history of present illness; the corresponding figures for the hypothermia therapy-alone group were 28.6% and 57.1%. Major complications are listed in Table 2. There was no sepsis as a complication.

**Table 1:** Patient characteristics.

		Temcell combination group (N = 6)	Hypothermia therapy-alone group (N = 7)
Gestational weeks at delivery (weeks)	mean ± SD	38.88 ± 1.04	40.22 ± 0.88
Birth weight (g)	mean ± SD	2900.8 ± 415.3	3197.7 ± 286.5
Age of mother (years)	mean ± SD	32.8 ± 3.2	33.7 ± 3.4
Core body temperature on arrival at the clinical site (°C)	n	6	6
	mean ± SD	35.62 ± 1.40	36.83 ± 0.52
Sex, n (%)	Male	2 (33.3)	3 (42.9)
	Female	4 (66.7)	4 (57.1)
Sarnat classification, n (%)	Stage 1	0 (0.0)	0 (0.0)
	Stage 2	5 (83.3)	6 (85.7)
	Stage 3	1 (16.7)	1 (14.3)
Transported from another hospital, n (%)	−	2 (33.3)	1 (14.3)
	+	4 (66.7)	6 (85.7)
Neonatal resuscitation, n (%)		6 (100.0)	7 (100.0)
Sentinel event, n (%)	−	1 (16.7)	3 (42.9)
	+	5 (83.3)	4 (57.1)
Medication status during pregnancy, n (%)	−	0 (0.0)	1 (14.3)
	+	6 (100.0)	6 (85.7)
Complications, n (%)	−	1 (16.7)	0 (0.0)
	+	5 (83.3)	7 (100.0)

SD: standard deviation.

**Table 2:** Individual patient characteristics.

ID	Group	Sex	Gestational weeks at delivery (weeks)	Birth weight (g)	Blood gas analysis (within 60 min after birth)			Sarnat classification	Complications
					pO_2_ (mmHg)	pH	Base deficit (mmol/L)		
01–01	A	F	39.9	3502	62.8	6.8	− 27.1	Stage 2	Anaemia, Coagulation disorder neonatal, Acidosis, Subgaleal haematoma, Subdural haematoma
02–01	A	F	37.1	2522	21.8	6.8	− 26.2	Stage 2	Hypocalcaemia, Hyponatraemia
02–02	A	F	38.1	2825	3.6	6.6	− 33.4	Stage 2	Hypovolaemia, Myocardial ischaemia, Urinary retention, Congenital hydronephrosis
04–01	B	F	41.1	3026	6.7	6.7	− 25.3	Stage 3	Cardiac failure, Meconium aspiration syndrome, Oliguria, Cephalhaematoma, C-reactive protein increased
05–01	A	M	40.7	2752	11.4	7.0	− 17.1	Stage 2	Haemoglobin increased, Neutrophil percentage abnormal
05–02	B	M	40.4	3376	48.7	7.5	− 3.3	Stage 2	Meconium aspiration syndrome
06–01	B	F	40.9	3532	28.7	6.8	− 21.9	Stage 2	Anaemia, Disseminated intravascular coagulation in newborn, Adrenal insufficiency, Metabolic acidosis, Neonatal respiratory distress syndrome, Renal disorder
07–01	B	F	40.9	3176	ND	ND	ND	Stage 2	Coagulation disorder neonatal, Circulatory failure neonatal, Neonatal respiratory distress syndrome, Pulmonary haemorrhage neonatal, Renal tubular necrosis
07–02	A	F	39.7	2386	26.5	6.9	− 22.4	Stage 3	Disseminated intravascular coagulation, Prerenal failure, Newborn persistent pulmonary hypertension
08–01	B	M	39.9	3524	214.0	7.2	− 14.1	Stage 2	Anaemia, Cephalhaematoma
08–02	B	F	38.6	2922	107.0	6.9	− 25.9	Stage 2	Hypoglycaemia, Urinary retention
09–01	A	M	39.1	2988	66.0	6.9	− 27.0	Stage 2	ND
09–02	B	M	39.9	2828	51.0	6.8	− 22.0	Stage 2	Meconium aspiration syndrome, Neonatal pneumothorax, Newborn persistent pulmonary hypertension
10–01	A	M	39.3	3182	20.9	6.8	− 24.8	Stage 2	Hypokalaemia, Hypotension, Oedema

A: Temcell combination group; B: Hypothermia therapy-alone group.

F: Female; M: Male; ND: no data.

Neurocognitive evaluation with the KSPD showed that 66.7% (4/6) of the patients in the Temcell combination group and 57.1% (4/7) of those in the hypothermia therapy-alone group were responsive to treatment (Table 3). In subsequent evaluation of each patient by the independent case review committee, 66.7% (4/6) of the Temcell group patients and 71.4% (5/7) of those in the hypothermia therapy-alone group were judged to have responded to treatment. One patient in the hypothermia therapy-alone group who was initially judged as non-responsive was later upgraded to responsive because of his overall developmental quotient of 84 at 18 months. Even though this was lower than his score at 12 months of 97, taken together with his mostly normal scores on BSID-III, it was taken to represent fairly well-preserved neurocognitive development. The mean developmental quotients at age 12 and 18 months were 98.7 and 91.0 in the Temcell combination group, and 86.0 and 88.4 in the hypothermia therapy-alone group (Table 3).

**Table 3:** Overall developmental quotients as assessed with the Kyoto Scale of Psychological Development 2001 and the Bayley Scales of Infant Development, Third edition.

			Temcell combination group (N = 6)	Hypothermia therapy-alone group (N = 7)
Effective (overall developmental quotient in KSPD ≥ 85 at 18 months) (n/N, %)			4/6 (66.7)	4/7 (57.1)
KSPD	12 months	n	3	6
		mean ± SD	98.7 ± 5.5	86.0 ± 25.3
	18 months	n	5	7
		mean ± SD	91.0 ± 9.5	88.4 ± 31.6
BSID-III at 18 months	Cognitive	n	5	7
		mean ± SD	88.8 ± 13.1	85.4 ± 30.9
	Receptive communication	n	5	7
		mean ± SD	67.6 ± 27.0	76.6 ± 33.6
	Expressive communication	n	5	7
		mean ± SD	68.8 ± 1.1	69.4 ± 26.8
	Fine motor	n	5	7
		mean ± SD	100.6 ± 14.5	96.7 ± 36.3
	Gross motor	n	5	7
		mean ± SD	87.2 ± 15.5	75.6 ± 29.7

BSID-III: Bayley Scales of Infant Development, Third edition; KSPD: Kyoto Scale of Psychological Development 2001; SD: standard deviation.

Brain MRI findings at 18 months of age in the Temcell combination group were normal (grade 0) in 4 patients, grade 2A in 1, and missing in 1; in the hypothermia therapy-alone group, they were normal in 6 patients and grade 3 in 1 patient (Table 4).

**Table 4:** Brain MRI and GMFCS.

	Temcell combination group (N = 6)		Hypothermia therapy-alone group (N = 7)	
Brain MRI (grade)	Day10	18 months	Day10	18 months
0	3	4	5	6
1A	1	0	1	0
2A	0	1	0	0
2B	1	0	1	0
3	1	0	0	1
Missing	0	1	0	0
GMFCS (level)	12 months	18 months	12 months	18 months
I	5	5	3	6
II	0	0	2	0
III	0	0	1	0
V	0	0	1	1
Missing	1	1	0	0

On GMFCS, 5 patients in the Temcell combination group were classified as level I at 18 months of age. Apart from 1 patient on whom we lacked data, all patients showed no change from the classification at 12 months of age. In the hypothermia therapy-alone group, 6 patients were level I, and 1 was level V. Of the 6 level I patients, 2 had improved from level II at 12 months, and 1 had improved from level III; the 1 level V patient remained unchanged (Table 4).

The mean total Thompson scores on postnatal days 1, 2, and 3 were 11.3, 11.8, and 10.5, respectively, in the Temcell combination group, and 12.0, 11.4, and 8.0, respectively, in the hypothermia therapy alone-group. The results of other secondary endpoints are summarized in Supplementary Table S1.

AEs occurred in all 7 patients in each group (65 events in the Temcell combination group, and 60 in the hypothermia therapy-alone group) (Supplementary Table S2), but none were severe, and there were no deaths. Three moderate AEs, i.e. adrenal insufficiency, diabetes insipidus and tibial fracture, occurred in 2 patients (28.6%) in the Temcell combination group, but all other AEs were mild in severity. The AEs observed in more than 3 patients in the combination therapy group were diaper rash (n = 5) and infantile eczema (n = 4), while those in the hypothermia therapy-alone group were diaper rash (n = 4), and laryngitis (n = 3). Three adverse drug reactions occurred in 3 patients (42.9%) in the Temcell combination group (Supplementary Table S2): supraventricular extrasystoles, hypotension, and cholestasis in 1 patient (14.3%) each. All of these were mild in severity and resolved during the study period. Abnormal laboratory data registered as AE in the Temcell combination group were iron-deficiency anaemia, low albuminemia, hypokalemia, hyperglycemia and hypoglycemia (n = 1, respectively), while those in the hypothermia therapy-alone group were increased C-reactive protein (n = 2), iron-deficiency anemia, low albuminemia, hypokalemia, hyperuricemia, increased plasma alkaline phosphatase, hyperbilirubinemia (n = 1, respectively). One patient in the hypothermia therapy-alone group had epileptic seizures (symptomatic focal epilepsy) at 12 and 18 months of age, and 1 patient in the Temcell combination group received respiratory support with a tracheostomy at 7 months of age and had a gastrostomy at 12 months of age; the general condition of this patient was poor, and he required support in eating, moving, dressing, and excretion. No patients in either group used inotropic agents. Percutaneous oxygen saturation and the results of other safety endpoints are given in Supplementary Table S3 and S4, respectively.

In summary, both safety and efficacy evaluation in the study found no marked differences between the Temcell combination group and the hypothermia therapy-alone group.

## Discussion

Great efforts have been made to address the severe and often fatal CNS damage caused by nHIE, in particular through the application of therapeutic hypothermia. The immunomodulatory effects of MSCs have been shown preclinically to be beneficial in treating ischemic lesions in the CNS^6–9^, and treatment of nHIE patients with a combination of MSCs and therapeutic hypothermia has been reported to be superior to hypothermia alone in patients with nHIE^13^. Likewise, Temcell, a product of hMSCs isolated from bone marrow fluid, can be expected to potentially limit the CNS damages in patients with nHIE by suppressing the inflammatory reactions associated with hypoxia and ischemia/reperfusion.

This article reports the results of the first randomized trial of Temcell designed to evaluate its safety and efficacy in combination with therapeutic hypothermia in treating nHIE. In terms of safety, while AEs were observed in all of the patients undergoing the combination treatment, all of the events were mild or moderate in severity and the incidence rates were similar to those in the group of patients receiving therapeutic hypothermia alone, suggesting that the safety profile of Temcell for patients with nHIE is comparable to that of therapeutic hypothermia.

Regarding efficacy evaluation, the number of patients who showed observable treatment response was similar in the Temcell combination group and the hypothermia therapy-alone group. This may be attributable to the high response rate among the patients undergoing hypothermia therapy alone, in which whole-body cooling was used. Whole-body cooling is the mainstream hypothermia therapy at present^24^, and has been shown to have a better, or at least similar, neuroprotective effect in patients with HIE when compared with selective cranial cooling^25^. The response rate in our hypothermia therapy-alone group was actually just as high as that in the Temcell combination therapy group and, despite the limited sample size, the fact that more than 60% of the patients had overall developmental quotient scores of 85 or higher (final overall efficacy assessment by the independent case review committee) further underscores the benefits of hypothermia therapy for patients with nHIE.

Limitations of the present study include its small sample size, which was not amenable to robust efficacy evaluation of Temcell, and the primary endpoints not being met by the study results. The steroid premedication for 5 of the 6 patients in the Temcell combination group as considered necessary by the investigators, as opposed to none in the hypothermia therapy-alone group, may constitute an additional confounding factor for efficacy and safety comparison between the two groups. Furthermore, the marked heterogeneity of nHIE itself, as shown by the enormous complexity of its clinical picture and multifaceted underlying factors and various complications, may make it inimical to efficacy and safety evaluation in a clinical trial involving a comparable homogenous patient population. In fact, any attempt to evaluate helpful new treatments is probably extremely difficult. Given this dilemma, even somewhat equivocal data from a small study may be informative and well worth sharing. A further trial, with a larger sample size and measures in place to control the above-mentioned heterogeneity as far as possible, would help illustrate more clearly the efficacy of MSCs for the treatment of CNS damages in patients with nHIE.

In summary, this exploratory study has shown that Temcell administration in combination with therapeutic hypothermia has acceptable safety and tolerability levels for use by patients with nHIE. The preliminary findings of this study are significant in that they provide further evidence of the clinical benefits of therapeutic hypothermia with whole-body cooling for patients with nHIE.

## Electronic supplementary material

Below is the link to the electronic supplementary material.

Supplementary Material 1

## Data Availability

The datasets used or analyzed during the current study are available from the corresponding author upon reasonable request.
