# Supplementary material for: Combined treatment with mesenchymal stem cells and therapeutic hypothermia for neonatal hypoxic ischemic encephalopathy: a phase 1/2 randomized trial
Source: Sci Rep. 2025 May 10;15:16302. doi: 10.1038/s41598-025-98504-z (PMC12065831; doi:10.1038/s41598-025-98504-z)
Supplement: Supplementary file 1 — Supplementary Material 1 [file 41598_2025_98504_MOESM1_ESM.docx]

Supplementary Table S1. Other secondary endpoints

|  | **Temcell combination group (N = 6)** | | | **Hypothermia therapy-alone group (N = 7)** | | |
| --- | --- | --- | --- | --- | --- | --- |
|  | 7 months | 12 months | 18 months | 7 months | 12 months | 18 months |
| Epileptic seizures, n (%) | 0 (0.0) | 0 (0.0) | 0 (0.0) | 0 (0.0) | 1 (14.3) | 1 (14.3) |
| Gastrostomy, n (%) | 0 (0.0) | 1 (16.7) | 1 (16.7) | 0 (0.0) | 0 (0.0) | 0 (0.0) |
| Ventilation with tracheostomy, n (%) | 1 (16.7) | 1 (16.7) | 1 (16.7) | 0 (0.0) | 0 (0.0) | 0 (0.0) |
| Use of inotropic agents, n (%) | 0 (0.0) | 0 (0.0) | 0 (0.0) | 0 (0.0) | 0 (0.0) | 0 (0.0) |
| Appetite, n (%) | 5 (83.3) | 5 (83.3) | 5 (83.3) | 6 (85.7) | 6 (85.7) | 7 (100.0) |
| Need for physical support, n (%) | 1 (16.7) | 1 (16.7) | 1 (16.7) | 0 (0.0) | 0 (0.0) | 0 (0.0) |

Supplementary Table S2. Brief summary of adverse events

|  | | **Temcell combination group (N = 7)** | | **Hypothermia therapy-alone group (N = 7)** | |
| --- | --- | --- | --- | --- | --- |
|  |  | **AEs** | **n (%)** | **AEs** | **n (%)** |
| Adverse events | | 65 | 7 (100.0) | 60 | 7 (100.0) |
| Severity | Mild | 62 | 7 (100.0) | 60 | 7 (100.0) |
|  | Moderate | 3 | 2 (28.6) | 0 | 0 (0.0) |
| Adverse drug reactions | | 3 | 3 (42.9) | NA | NA |
| Serious adverse events | | 0 | 0 (0.0) | 0 | 0 (0.0) |
| Fatal adverse events | | 0 | 0 (0.0) | 0 | 0 (0.0) |

AE: adverse event, NA: not applicable

Adverse drug reactions denote those AEs which are considered to be definitely related to the study drug itself in terms of causality, thus counted as AE as well.

The adverse events observed in more than 3 patients were diaper rash and infantile eczema in Temcell combination group, and diaper rash and laryngitis in hypothermia therapy-alone group.

The observed moderate AEs were adrenal insufficiency, diabetes insipidus, and tibial fracture.

Supplementary Table S3. Percutaneous oxygen saturation before and after Temcell administration (Temcell combination group; N = 7)

| Percutaneous oxygen saturation | | Day 0 | Day 4 | Day 7 | Day 10 | Day 13 | Day 16 | Day 19 | Day 22 |
| --- | --- | --- | --- | --- | --- | --- | --- | --- | --- |
| Pre administration | n | 7 | 7 | 6 | 6 | 6 | 5 | 6 | 6 |
|  | mean ± SD | 97.1 ± 3.8 | 97.4 ± 1.4 | 97.7 ± 1.8 | 96.8 ± 2.1 | 98.0 ± 1.8 | 98.4 ± 1.8 | 98.8 ± 1.8 | 99.2 ± 1.3 |
| Post administration | n | 6 | 7 | 6 | 6 | 6 | 6 | 5 | 6 |
|  | mean ± SD | 97.8 ± 2.2 | 97.9 ± 2.3 | 96.7 ± 3.1 | 96.5 ± 4.5 | 97.5 ± 1.6 | 99.0 ± 1.5 | 98.8 ± 1.1 | 98.7 ± 1.5 |

Supplementary Table S4. Other safety endpoints

|  | **Temcell combination group (N = 7)** | | | **Hypothermia therapy-alone group (N = 7)** | | |
| --- | --- | --- | --- | --- | --- | --- |
|  | Baseline | Day 4 | 3 months | Baseline | Day 4 | 3 months |
| Abnormality in X-ray, n (%) | 0 (0.0) | 1 (14.3) | 1 (14.3) | 3 (42.9) | 0 (0.0) | 0 (0.0) |
| Abnormality in echocardiogram, n (%) | 0 (0.0) | 0 (0.0) | 0 (0.0) | 4 (57.1) | 3 (42.9) | 0 (0.0) |
| Abnormality in echoencephalogram, n (%) | 1 (14.3) | 1 (14.3) | 1 (14.3) | 1 (14.3) | 1 (14.3) | 0 (0.0) |
